# Integration of Transcriptome and Proteome in Lymph Nodes Reveal the Different Immune Responses to PRRSV Between PRRSV-Resistant Tongcheng Pigs and PRRSV-Susceptible Large White Pigs

**DOI:** 10.3389/fgene.2022.800178

**Published:** 2022-01-27

**Authors:** Wan Liang, Xiangge Meng, Yueran Zhen, Yu Zhang, Xueying Hu, Qingde Zhang, Xiang Zhou, Bang Liu

**Affiliations:** ^1^ Key Laboratory of Agricultural Animal Genetics, Breeding and Reproduction of Ministry of Education, College of Animal Science and Technology, Huazhong Agricultural University, Wuhan, China; ^2^ Key Laboratory of Prevention and Control Agents for Animal Bacteriosis (Ministry of Agriculture), Animal Husbandry and Veterinary Institute, Hubei Academy of Agricultural Science, Wuhan, China; ^3^ College of Veterinary Medicine, Huazhong Agricultural University, Wuhan, China; ^4^ Laboratory Animal Center, College of Animal Science and Technology and Veterinary Medicine, Huazhong Agricultural University, Wuhan, China

**Keywords:** PRRSV, lymph node, transcriptome, proteome, Tongcheng pigs

## Abstract

Porcine reproductive and respiratory syndrome (PRRS) is an infectious disease that seriously affects the swine industry worldwide. Understanding the interaction between the host immune response and PRRS virus (PRRSV) can provide insight into the PRRSV pathogenesis, as well as potential clues to control PRRSV infection. Here, we examined the transcriptome and proteome differences of lymph nodes between PRRSV-resistant Tongcheng (TC) pigs and PRRSV-susceptible Large White (LW) pigs in response to PRRSV infection. 2245 and 1839 differentially expressed genes (DEGs) were detected in TC and LW pigs upon PRRSV infection, respectively. Transcriptome analysis revealed genetic differences in antigen presentation and metabolism between TC pigs and LW pigs, which may lead to different immune responses to PRRSV infection. Furthermore, 678 and 1000 differentially expressed proteins (DEPs) were identified in TC and LW pigs, and DEPs were mainly enriched in the metabolism pathways. Integrated analysis of transcriptome and proteome datasets revealed antigen recognition capacity, immune activation, cell cycles, and cell metabolism are important for PRRSV clearance. In conclusion, this study provides important resources on transcriptomic and proteomic levels in lymph nodes for further revealing the interaction between the host immune response and PRRSV, which would give us new insight into molecular mechanisms related to genetic complexity against PRRSV.

## Introduction

Porcine reproductive and respiratory syndrome (PRRS), caused by the PRRS virus (PRRSV), is a noticeable infectious and epidemic disease, with symptoms of reproductive failure in sows, respiratory disorders in pigs of all ages, and high mortality in young piglets (Ko et al., 2015). After being discovered in the United States in 1987 and Europe in 1990, PRRS quickly became pandemic in many countries (Rossow, 1998), and its outbreak and ongoing epidemic caused tremendous economic loss annually. The economic disease model indicated that PRRS might annually cause losses ranging from a median of €650 per sow in a severely affected farm (Nathues et al., 2017).

PRRSV is an enveloped, single-stranded positive-sense RNA virus belonging to the Arteriviridae family. PRRSV strains have been divided into two main genotypes (North American genotype and European genotype) (Dea et al., 2000). The target cells of PRRSV are porcine alveolar macrophages (PAMs) and macrophages in other tissues (Dokland, 2010; W. et al., 2010). Increasing evidence showed that PRRSV develops different strategies to evade innate and adaptive immune responses (Yoo et al., 2010; Huang et al., 2015). The adaptive immune response is critical to developing protective immunity against PRRSV (Michael and Michael, 2017). Notably, the severity of clinical symptoms in PRRSV-infected pigs significantly correlated with the intensity of cell-mediated immune responses (Lowe et al., 2005). However, the cell-mediated immune response against PRRSV is always delayed and defective due to reducing developing and circling T lymphocyte (T cell) populations in pigs during PRRSV infection (Dwivedi et al., 2012). Lymph nodes are the secondary lymphoid organs that are important to maintaining T cell pools. PRRSV can replicate in lymphoid organs and cause lymph nodes lesions, which affect the host’s cell-mediated immune response to PRRSV (Lamontagne et al., 2003). Furthermore, the down-regulated of major histocompatibility complex (MHC) class I and class II genes of antigen-presenting cells (APCs) during PRRSV infection would significantly affect antigen presentation and T cells activation (Chang et al., 2008; Che et al., 2011). Therefore, the activation of cell-mediated immune response could restrict PRRSV replication and reduce clinical diseases.

Although vaccination is one of the common strategies to control PRRS, it has only a partial protective effect because of the immune evasion and the highly variable RNA genome of PRRSV (Shi et al., 2010). The exploration of genetic improvements has provided a new feasible way for controlling PRRS (Rowland et al., 2012). A lot of studies have found that different breeds of pigs respond differently to PRRSV infection. Vincent and others observed that monocyte-derived macrophages (MDMs) from Large White (LW) pigs were more susceptible to PRRSV infection than MDMs from Duroc-Pietrain synthetic line (Vincent et al., 2005). Ait-Ali and others found that viral loads of PAMs from Landrace were lower than other breeds (Ait-Ali et al., 2007). Our previous study has found that Chinese indigenous Tongcheng (TC) pigs strongly resist PRRSV infection (Liang et al., 2016). TC pigs showed less severe clinical symptoms and a lower viremia level than LW pigs in response to PRRSV infection (Liang et al., 2016). TC pigs could promote the extravasation and migration of leukocytes and suppress apoptosis of the infected macrophages during PRRSV infection, and reduce the lung lesions (Liang et al., 2017). Furthermore, IFN-γ is a crucial biomarker for T cell-mediated cellular immune response. TC pigs had higher serum IFN-γ levels than LW pigs in response to PRRSV infection (Liang et al., 2016).

To understand the molecular mechanism of phenotypes related to resistance or tolerance to PRRSV in TC pigs, we performed transcriptome and proteome analysis of the inguinal lymph nodes (ILNs). Integrated analysis of transcriptome and proteome datasets revealed the critical pathways of resistance or tolerance to PRRSV in TC pigs, which gives us new insight into the molecular mechanisms of host genetic complexity against PRRSV.

## Materials and Methods

### Animal Experiment and Sample Collection

A total of twelve 5-week-old piglets (3 TC pigs and 3 LW pigs infected with highly pathogenic PRRSV WuH3 strain as PRRSV infected group, 3 TC pigs and 3 LW pigs injected with RPMI-1640 as control group) from our previous artificial challenge experiment were used in this study (Liang et al., 2016; Liang et al., 2017). Briefly, the PRRSV infected groups were intramuscularly challenged with highly pathogenic PRRSV WuH3 strain at a dose of 10^5^ CCID_50_/mL (3 ml/15 kg), and the control groups were intramuscularly challenged with the same amount of RPMI-1640 (Gibco, Grand Island, NY, United States). The viremia reached the peak value before days 7 post challenge (dpc), and significant lung lesion differences could be observed between TC pigs and LW pigs at 7 dpc (Zhou et al., 2011; Liang et al., 2016). As a result, all pigs were humanely euthanized for sampling at 7 dpc, and the ILNs were collected for the following transcriptomic and proteomic profiling.

All animal procedures were approved by the Ethical Committee for Animal Experiments at Huazhong Agricultural University, Wuhan, China. The animal experiments were performed at the Laboratory Animal Center of Huazhong Agricultural University (Animal experiment approval No. HZAUSW-2013-005).

### RNA and Protein Preparation

The ILNs saved in −80°C were used for RNA and protein preparation. The total RNA of ILNs was extracted with Trizol reagent (Invitrogen, Carlsbad, CA, United States) according to the manufacturer’s protocol. RNA degradation and contamination were monitored on 1% agarose gels and Nano Photometer spectrophotometer (IMPLEN, CA, United States). RNA concentration and integrity were measured with Qubit RNA Assay Kit (Life Technologies, CA, United States) and RNA Nano 6000 Assay Kit (Agilent Technologies, CA, United States).

The ILNs mixed with lysis buffer and magnetic beads were lysed to release total protein. Then, the supernatant was processed with dithiothreitol and iodoacetamide, and quantified after centrifugation. The concentration and integrity of total protein were measured with Bradford assay and SDS-PAGE according to the manufacturer’s protocol.

### RNA Sequencing and Data Analysis

The qualified total RNA was used to generate transcriptome sequencing libraries using NEBNext^®^ Ultra^TM^ RNA Library Prep Kit for Illumina^®^ (NEB, United States) following the manufacturer’s recommendations. The quality of sequencing libraries was assessed on the Agilent Bioanalyzer 2100 system. After cluster generation, the libraries were sequenced on an Illumina Hiseq 2000 platform, and 150 bp paired-end reads were obtained. The ILNs transcriptome datasets were composed of 12 samples from four groups, including two control groups (TC-Control and LW-Control) and two PRRSV-infected groups (TC-Infection and LW-Infection). Each group included three individuals. After data cleaning, Hisat2 (Kim et al., 2015) was used to align the clean reads against the *Sus Scrofa 11.1* reference genome. The unique aligned reads were calculated with HTSeq (Simon et al., 2015) and determined the total number of reads at the gene level guided by the corresponding genome annotation. Differential expression analysis was conducted by DESeq2 (Love et al., 2014) R package (4.1.0). The genes with false discovery rate (FDR) < 0.01 and log_2_ (fold change) ≥1 were assigned as significantly differentially expressed genes (DEGs).

### Proteome Quantification and Data Analysis

The qualified total protein was digested to generate the peptides. After Isobaric tags for relative and absolute quantitation (iTRAQ) labeling and peptide fractionation, the peptides were separated by high performance liquid chromatography (HPLC). Mass spectrometer (MS) detection was performed with a Triple TOF 5600 System (SCIEX, Framingham, MA, United States) equipped with a Nanospray III source (SCIEX, Framingham, MA, United States), a pulled quartz tip as the emitter (New Objectives, Woburn, MA) and controlled with software Analyst 1.6 (AB SCIEX, Concord, ON). The MS data were searched against the Ensembl *Sus scrofa* database (http://ftp.ensembl.org/pub/release-104/fasta/sus_scrofa/). IQuant (Wen et al., 2015) was employed to quantitatively analyze the labeled peptides with isobaric tags. For protein quantitation, it was required that a protein contains at least one unique peptide segment. The protein with a *q*-value < 0.05 and fold change >1.20 or <0.83 in at least two of the three replicates, and the mean fold change of three replicates >1.20 or <0.83 were assigned as significantly differentially expressed proteins (DEPs).

### Correlation Analysis of Transcriptome and Proteome Datasets

The Spearman rank correlations (r) and corresponding *p*-value were calculated using the cor.test function in the statistics package R. The log_2_ (fold change) of mRNA transcripts and their corresponding proteins were defined as the correlation analysis parameters. GraphPad Prism 9 (San Diego, CA, United States) was used to draw quadrant maps of transcripts and proteins of differential expression.

### GO Terms and KEGG Pathway Analysis

The Gene Ontology (GO) annotation and pathway enrichment analysis of the DEGs or DEPs between groups were performed with DAVID (https://david.ncifcrf.gov/). A *p*-value < 0.05 was considered to indicate the significance. The common enriched terms of GO and KEGG between transcriptome and proteome in ILNs were compared and integrated.

## Results

### Transcriptome Analysis of ILNs in Response to PRRSV Infection

**FIGURE 1:**
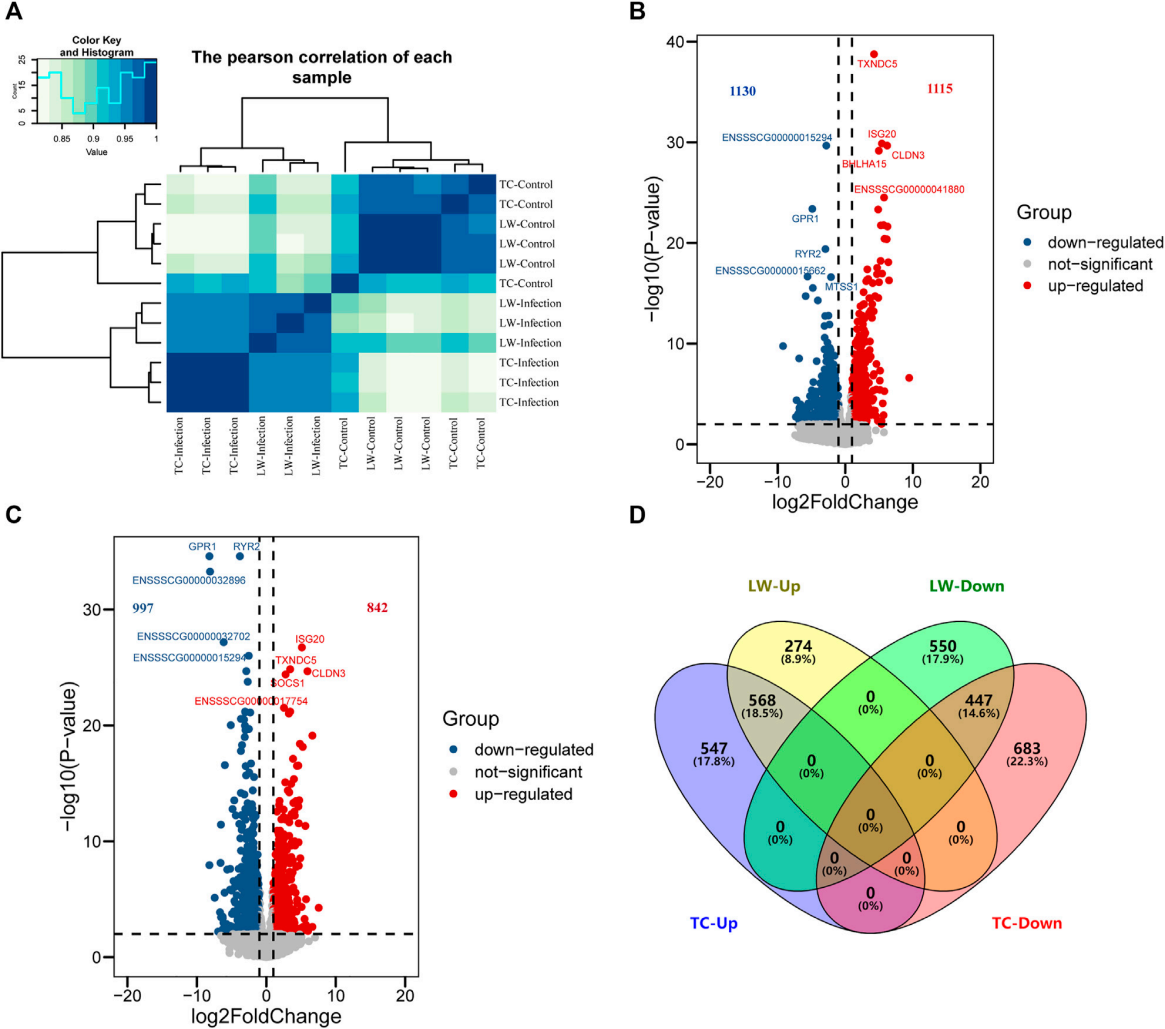
DEGs in TC and LW pigs in response to PRRSV infection. **(A)** Correlation of samples based on DEGs; **(B)** DEGs in ILNs of TC pig in response to PRRSV; **(C)** DEGs in ILNs of LW pig in response to PRRSV; **(D)** Venn diagram of DEGs in TC pigs and LW pigs.

After sequencing and raw data cleaning, each sample harvested more than 11G of high-quality data. Pearson correlation analysis showed a great difference between control and PRRSV-infected groups. In addition, the samples in the TC-Infection and LW-Infection groups could be well distinguished. However, there was no difference between TC pigs and LW pigs in the control groups (Figure 1A). Furthermore, we identified 2245 differentially expressed genes (DEGs) in TC pigs upon PRRSV infection (TC-Infection vs TC-Control), of which 1115 were up-regulated and 1130 were down-regulated (Figure 1B; Supplementary Table S1). For LW pigs, there were 1839 DEGs (LW-Infection vs LW-Control), of which 842 were up-regulated, and 997 were down-regulated (Figure 1C; Supplementary Table S1). Among the DEGs, there were 568 common up-regulated and 447 common down-regulated DEGs (Figure 1D). *TXNDC5* was the most significantly up-regulated gene in TC pigs (FDR = 1.76E-39, fold change = 19.10), which could encode disulfide isomerase and protect hypoxic cells from apoptosis (Figure 1B; Supplementary Table S1).

**FIGURE 2:**
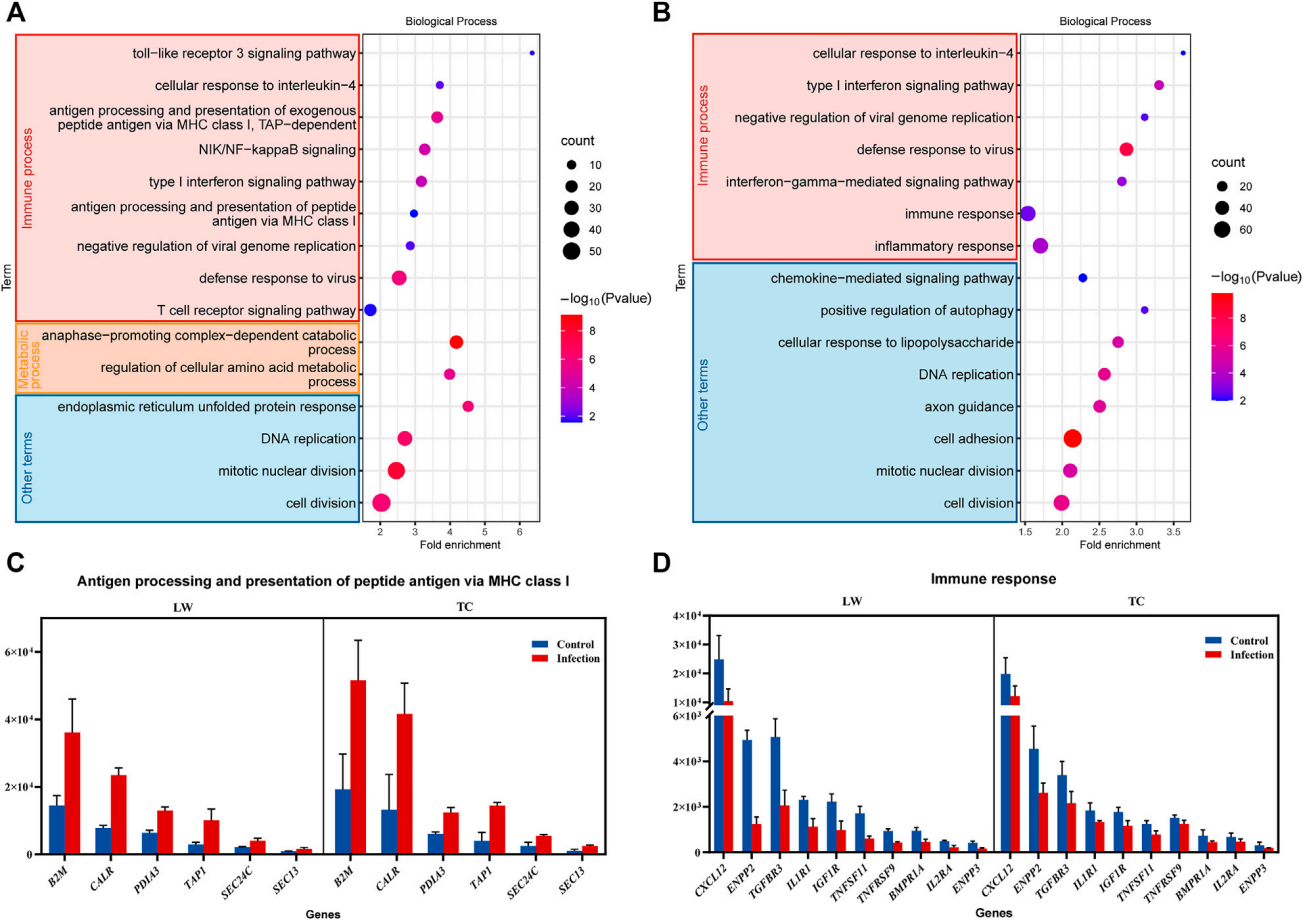
The gene ontology (GO) enrichment of the DEGs. **(A)** GO enrichment of the DEGs in TC pigs; **(B)** GO enrichment of the DEGs in LW pigs; **(C,D)** DEGs in different terms of GO enrichment.

The DEGs of the ILNs in TC pigs and LW pigs were uploaded to DAVID (https://david.ncifcrf.gov/) for functional pathway enrichment (Figure 2 and Supplementary Figure S2). In the enriched terms of KEGG, there were 8 common pathways in TC and LW pigs, and DEGs in TC pigs were perferred to be enriched in metabolic process, including biosynthesis of amino acids (hsa01230), carbon metabolism (hsa01200), N-glycan biosynthesis (hsa00510) and 2-oxocarboxylic acid metabolism (hsa01210). Then, we mainly focused on the GO biological process (BP).

The GO enriched terms of DEGs in TC pigs were mainly related to immune response processes, such as T cell receptor signaling pathway (GO:0050852), defense response to virus (GO:0051607), NIK/NF-κB signaling (GO:0038061), antigen processing and presentation of peptide antigen via MHC class I (GO:0002474) (Figure 2A). Besides, cell division (GO:0051301), regulation of cellular amino acid metabolic process (GO:0006521), and anaphase−promoting complex−dependent catabolic process (GO:0031145) were enriched (Figure 2A). Interestingly, a large number of genes were significantly up-regulated in TC pigs after PRRSV infection, which was correlated with antigen processing and presentation, including *B2M*, *CALR*, *PDLA3*, and *TAP1* in antigen processing and presentation of peptide antigen *via* MHC class I (Figure 2C), *PSMB8*, *PSMD2*, *PLCG1*, and *PSMB4* in T cell receptor signaling pathway (Supplementary Figure S1).

For LW pigs, the enriched terms of DEGs were associated with inflammatory response (GO:0006954), defense response to virus (GO:0051607), type I interferon signaling pathway (GO:0060337), DNA replication (GO:0006260), cellular response to lipopolysaccharide (GO:0071222) and cell adhesion (GO:0007155) (Figure 2B). Among the enriched terms, there was a clear difference between LW pigs and TC pigs. For LW pigs, the inflammatory response was enriched, and there was no enrichment in antigen processing and presentation. Meanwhile, there were more down-regulated DEGs (*CXCL12*, *ENPP2*, *IL1R1*, *IGF1R*) involved in immune response in LW pigs (Figure 2D), which meant that the immune responses in the ILNs of LW pigs were suppressed, and it was constant with our previous results that IL-10 in LW pigs were 2–8 times higher than TC pigs in blood during PRRSV infection (Liang et al., 2016).

### Proteome Analysis of ILNs in Response to PRRSV Infection

**FIGURE 3:**
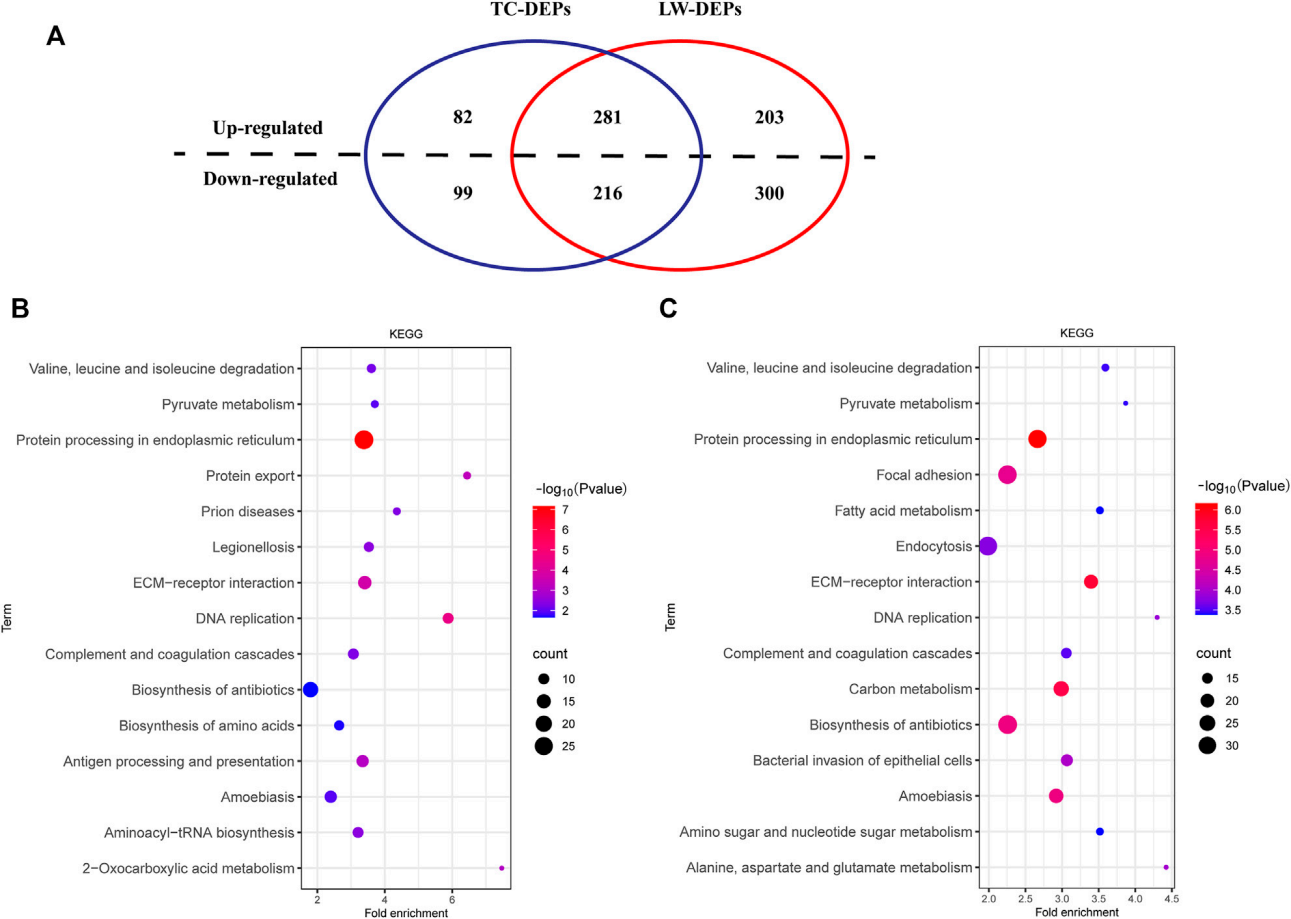
DEPs and KEGG enrichment. **(A)** Venn diagram of DEPs in TC and LW pigs after PRRSV challenging; **(B)** KEGG of the DEPs in TC pigs; **(C)** KEGG of the DEPs in LW pigs.

A total of 1031569 spectra and 55124 peptides were generated by the quantitation analysis of proteome data in ILNs. Finally, 6296 proteins were identified for subsequent analysis of differentially expressed proteins (DEPs) (Figure 3A; Supplementary Table S2). In ILNs of TC pigs, we identified 678 DEPs (TC-Infection vs TC-Control), including 363 up-regulated and 315 down-regulated DEPs. There were 484 up-regulated and 516 down-regulated DEPs in ILNs of LW pigs after PRRSV challenging (LW-Infection vs LW-Control) (Figure 3A; Supplementary Table S3). Among the DEPs, 281 common up-regulated, and 216 common down-regulated DEPs were identified in both TC and LW pigs (Figure 3A).

Then, we performed the enrichment analysis to further gain insights into the biological functions of DEPs in TC and LW pigs (Figures 3B,C; Supplementary Figure S3). Among the 15 most enriched pathways of KEGG, TC and LW pigs shared 8 common pathways, including protein processing in endoplasmic reticulum (hsa04141), DNA replication (hsa03030), ECM-receptor interaction (hsa04512), complement and coagulation cascades (hsa04610), amoebiasis (hsa05146), valine, leucine and isoleucine degradation (hsa00280), pyruvate metabolism (hsa00620) and biosynthesis of antibiotics (hsa01130). Besides, DEPs in TC pigs were enriched in the biosynthesis of amino acids (hsa01230), antigen processing and presentation (hsa04612), protein export (hsa03060), aminoacyl-tRNA biosynthesis (hsa00970), and DEPs in LW pigs were enriched in Focal adhesion (hsa04510), endocytosis (hsa04144), alanine, aspartate and glutamate metabolism (hsa00250). Notably, the DEPs in TC pigs were enriched in antigen processing and presentation, which was consistent with the transcriptome datasets. In the biological process, the response to virus (GO:0009615) and type I interferon signaling pathway (GO:0060337) were enriched in TC and LW pigs (Supplementary Figure S3).

Among the DEPs, ACO1, BCAT2, ASS1, GOT1, BCAT1, GOT2, SHMT2, MAT2A, and IDH2 were involved in the process of biosynthesis of amino acids, and BCAT1 was found up-regulated in TC pigs while down-regulated in LW pigs. In addition, the fold changes of down-regulated proteins in LW pigs were higher than that in TC pigs (Supplementary Figure S4A). The DEPs in Fc gamma R-mediated phagocytosis showed a similar tendency with genes in biosynthesis of amino acids (Supplementary Figure S4B), while the DEPs in the apoptotic process had the opposite trend (Supplementary Figure S4C), in which more up-regulated DEPs were observed in LW pigs, MX1, S100A8, C1QBP, and FXR1 were down-regulated in TC pigs and up-regulated in LW pigs.

### Comparative Analysis Between Protein Abundance and Gene Expression Levels of ILNs in TC and LW Pigs

**FIGURE 4:**
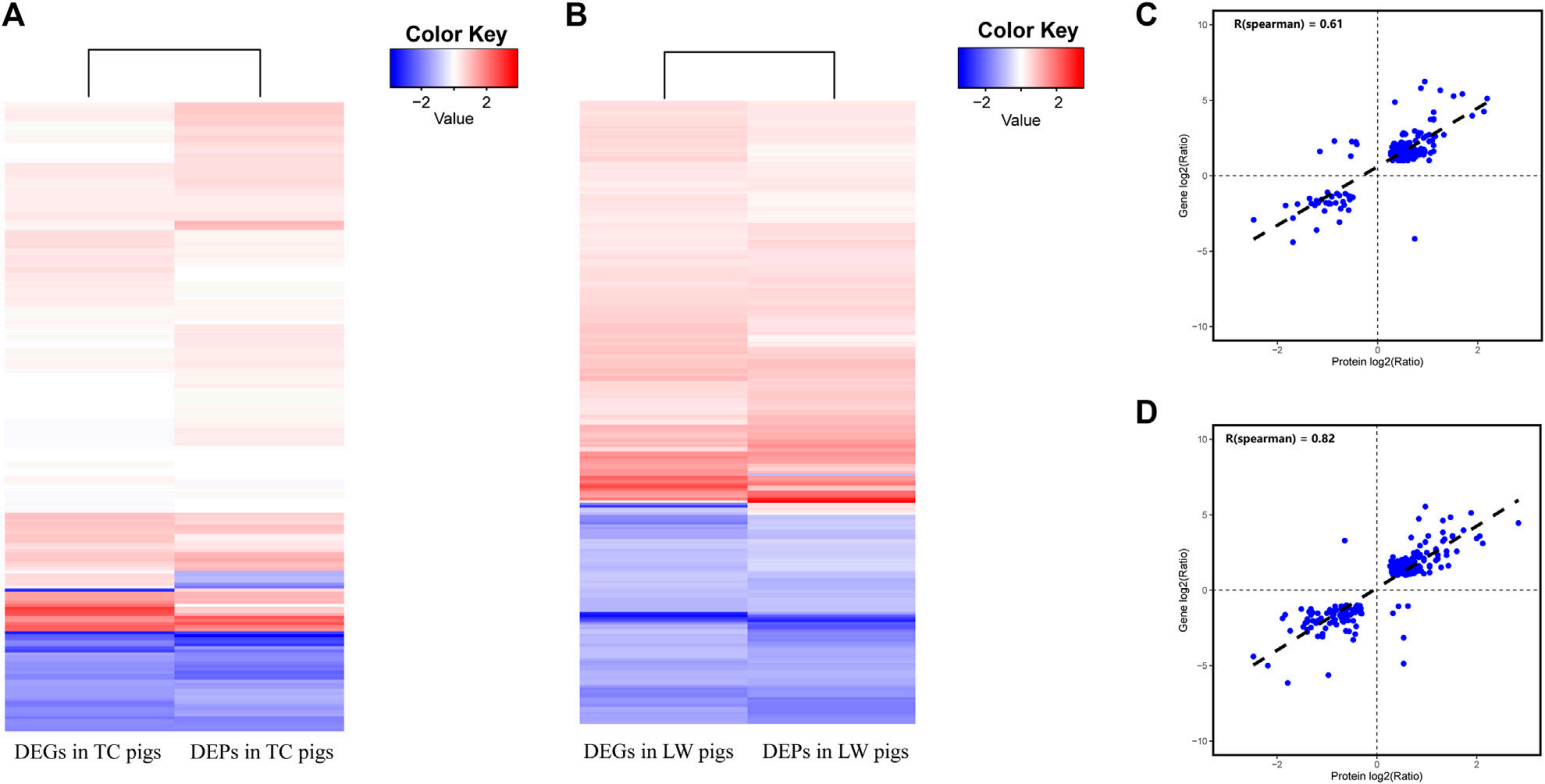
Comparison of protein abundance and gene expression levels in TC and LW pigs. **(A)** Comparison of DEGs and DEPs in TC pigs; **(B)** Comparison of DEGs and DEPs in LW pigs; **(C)** Correlations between DEGs and DEPs in TC pigs; **(D)** Correlations between DEGs and DEPs in LW pigs.

In this study, we performed the comparative analysis of transcriptome and proteome datasets in TC and LW pigs to provide a more accurate and comprehensive picture of gene expression profile. A total of 5482 genes and the encoded proteins were expressed in both transcriptome and proteome datasets (Supplementary Figure S5A). 207 genes belong to both DEGs and DEPs in TC pigs (Figure 4A; Supplementary Figure S5B), wherein 200 genes had the similar regulatory trends in both mRNA and protein levels. For example, 2′-5′-oligoadenylate synthetase like (OASL) and interferon alpha inducible protein 6 (IFI6) were significantly up-regulated in the TC-Infection group at both transcriptome and proteome datasets, which were involved in immune response during virus infection. In particular, OASL not only inhibits PRRSV replication but also activates interferon-β production (Wang et al., 2018). Furthermore, 256 genes belonging to both DEGs and DEPs in LW pigs (Figure 4B, Supplementary Figure S5C). Then correlation analysis was carried out to investigate the relationship between DEGs and DEPs in TC pigs and LW pigs. The Spearman correlation coefficient of TC pigs and LW pigs were 0.61 and 0.82, respectively, indicating that the expression trend of protein and mRNA were positively correlated (Figures 4C,D). Therefore, transcriptional regulation plays a major role when the pigs were stimulated by PRRSV.

### The integrative Analysis of Transcriptome and Proteome Revealed That the Antigen Recognition Capacity is Crucial for the Immune Activation of PRRSV Infection

**FIGURE 5:**
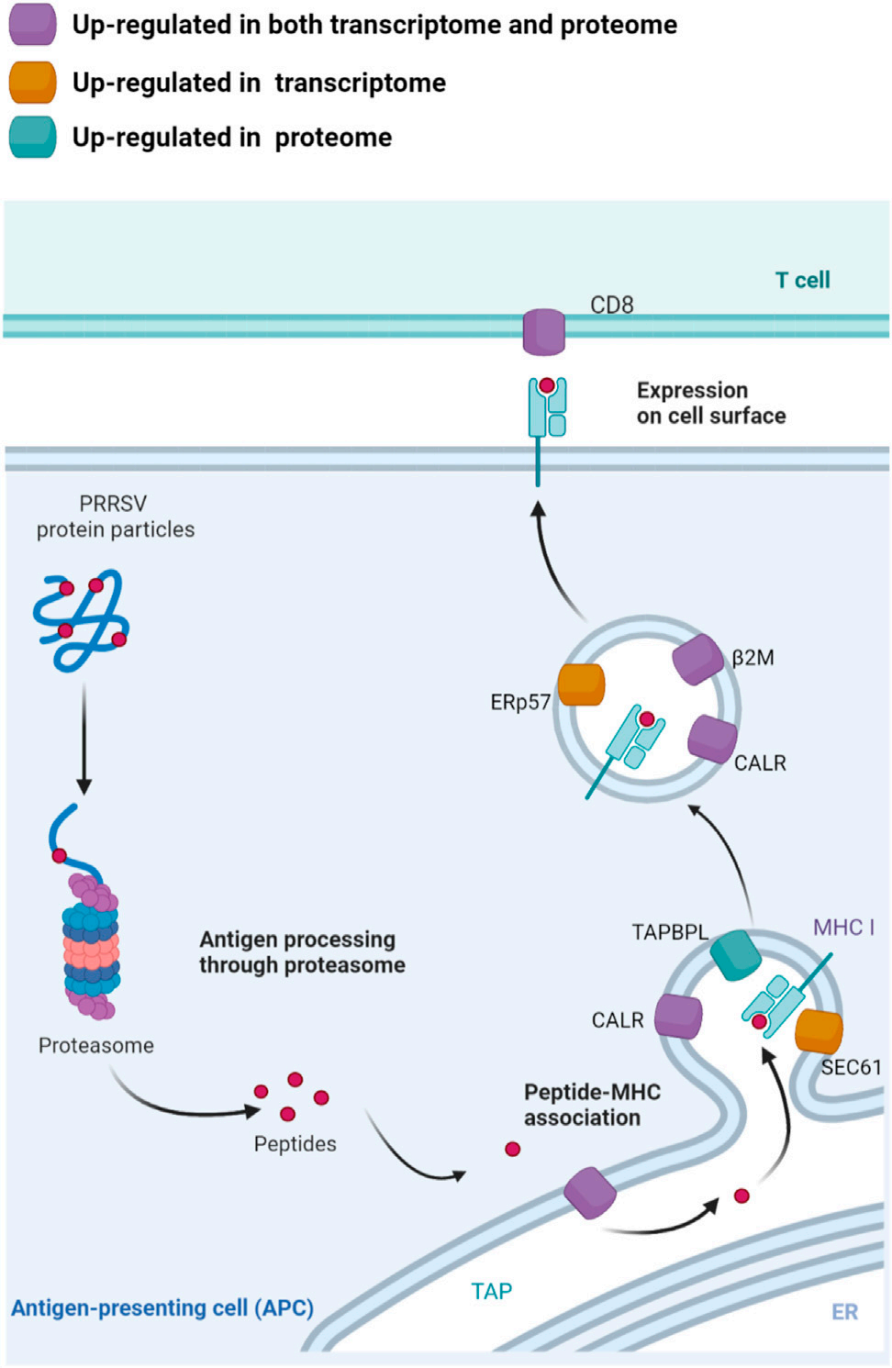
The integrated analysis of the mRNA and protein expression in TC pigs in the antigen processing and presentation. The up-regulated genes in the process of antigen transportation, degradation, assembly and presentation could contribute to antigen degradation and the recognition of peptides by T cells. Up-regulated in both of transcriptome and proteome datasets were marked with purple; Up-regulated in transcriptome datasets were marked with yellow; Up-regulated in proteome datasets were marked with green. The figure was created using BioRender.com.

The up-regulated genes in both transcriptome and proteome datasets of TC pigs were enriched in the pathway of antigen processing and presentation. As an endogenous antigen, PRRSV protein particles were first captured and degraded by immuno-proteasomes. The expression of proteasome 20S subunit beta 8 (PSMB8) and proteasome 20S subunit beta 9 (PSMB9) were up-regulated (Supplementary Figure S1), and several ATPases were up-regulated. In addition, the proteins responsibly for selective transport of antigen peptides to endoplasmic reticulum in TC pigs were up-regulated either in transcriptome datasets or in proteome datasets, including transporter associated with antigen processing (TAP), membrane proteins SEC61, calreticulin (CALR), TAP binding protein like (TAPBPL), endoplasmic reticulum resident protein 57 (ERp57), and microglobulin β2B (B2M) (Figure 5). When the antigen peptides were banded to MHC-I molecules, the MHC class I complexes would be expressed at the cell surface to activate the cellular immune response in TC pigs (Figure 5). Although the expression of MHC-II molecules was down-regulated in TC pigs, their expressions were still higher than that in LW pigs.

### The Integrative Analysis of Transcriptome and Proteome Revealed That Cell Cycles and Cell Metabolism Support the Immune Activation

**FIGURE 6:**
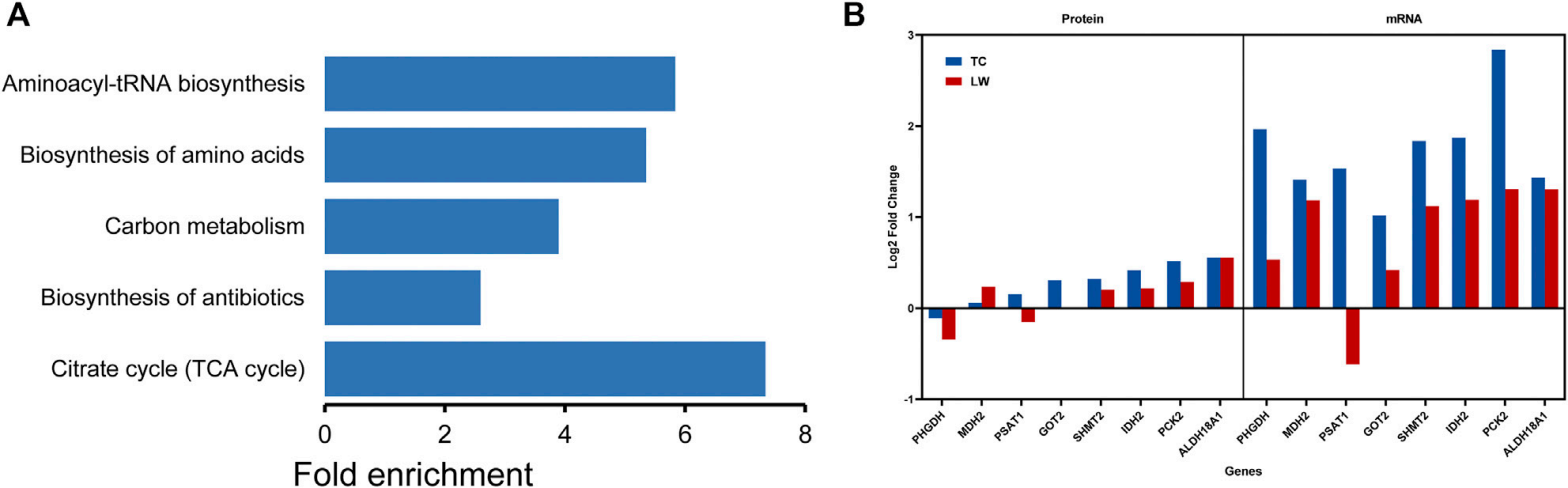
The integrated analysis of the mRNA and protein expression of the cell cycles and cell metabolism in TC pigs. **(A)** The main metabolism terms in TC pigs. **(B)** DEGs and DEPs in TCA cycle and carbon metabolism.

In TC pigs, many genes belonging to both DEGs and DEPs were enriched in the processes of cell cycles and cell metabolism, which would provide biological energy for cell activities and immune response (Figure 6A). In the Citrate cycle (TCA cycle) and carbon metabolism, *PSAT1*, *GOT2*, *SHMT2*, *IDH2*, *PCK2* in TC pigs showed a more prominent up-regulation trend than those in LW pigs in response to PRRSV infection (Figure 6B). Genes in biosynthesis of amino acids and aminoacyl-tRNA biosynthesis had the same trend, *GOT2*, *MAT2A*, *IDH2*, *HARS1*, *GARS1* were found up-regulated in TC pigs, and the fold changes of them in TC pigs were higher than that in LW pigs (Supplementary Figure S4A; Supplementary Tables S1, S3). The activation of these pathways would offer ATP for immune cell activation and material for the synthesis of RNA, DNA, proteins, and cell membrane. During the cell cycle signaling, *CHEK2*, *CDC26*, *CDK1*, *CDK2AP2*, *CCNB* were up-regulated in TC pigs (Supplementary Tables S1, S3). It reminded the cell cycle was activated after PRRSV infection in TC pigs.

## Discussion

PRRSV first infects and replicates in PAMs, then emigrates to different organs through blood and lymph circulation (Lunney et al., 2016). Notably, PRRSV infection results in lesions in lymph nodes, and all of PRRSV infected cells in the lymph nodes are macrophages (Duan et al., 1997). PRRSV escapes host-mediated clearance through hiding in the tissue macrophages, leading to a life-long subclinical infection (Bierk et al., 2001). In addition, the cellular immune response in response to PRRSV infection was suppressed due to acute lymphopenia in peripheral immune organs (Canelli et al., 2017). Therefore, the peripheral lymph nodes play an essential role in host resistance against PRRSV infection.

After PRRSV infection, the viral RNA and proteins of PRRSV are recognized by the host’s pattern-recognition receptors (PRRs), which then triggers the associated signaling pathways and the cellular immune response (Michael and Michael, 2017; Tqa et al., 2020). The activation of cellular immunity requires the coordinated action of multiple cell types. Antigen presenting cells, especially macrophages, are key regulators of initiating and regulating immune response and participate in T cell activation and effector function (Guerriero, 2019). Macrophages are the primary target cells of PRRSV, whose antigen presentation would be weakened after PRRSV infection (Rodríguez-Gómez et al., 2013; Stocks et al., 2018). In this study, the antigen presentation genes were higher expressed in lymph nodes of TC pigs, including *B2M*, *CALR*, *TAP1*, *PSMB8*, *PSMB9*, which made TC pigs have a better capacity to perform antigen presentation and induce stronger T-cell immune response. In addition, the transcriptome analysis also revealed that TC pigs have a higher expression level of MHC genes than LW pigs, which was consistent with the expression pattern in PAMs of TC pigs and LW pigs (Liang et al., 2017). These results suggested that PRRSV promotes the viral antigen presentation to cytotoxic T lymphocytes by increasing expression levels of genes in the antigen presentation process, which further enhanced the immune response of ILNs. In TC pigs, the activation of antigen presentation and T cell receptor signaling pathway may play a relevant role in the antiviral immune response.

The interaction and communication in immune cells coordinate host immune response to the invading pathogens. The innate immune response can stimulate and activate the adaptive immune response. The activated adaptive immune system also enhances innate immune response to the invading pathogens (Lsa et al., 2020; Mcdaniel et al., 2021). The activated T cells would up-regulate TNF receptor super family ligands including CD40L, which signal back to the APC to enhance the production of cytokines necessary for inflammation and immune responses (Mcdaniel et al., 2021). This is an advantageous strategy when the pathogens inhibit PRR signaling. In this study, the interferon regulatory factor family members *IRF4*, *IRF7*, and the IFN-stimulated genes (ISGs) including *ISG20*, *ISG12(A)*, *ISG15*, *IFIT1*, *IFIT5*, *IFITM3* *MX1*, *MX2*, and *OAS2* were up-regulated after PRRSV infection. IRF7 is the antagonistic target of PRRSV and plays an important role in the innate immune response against virus infection, which can regulate ISGs expression in the absence of IFN signaling (Ning et al., 2011; Chiang and Liu, 2018). Furthermore, some ISGs are directly induced by viruses and play an important role in maintaining the stability of the cell-virus environment (Sen and Peters, 2007; Schoggins and Rice, 2011). In TC pigs, the NIK/NF-κB signaling pathway and IFN-γ pathway were activated to regulate the expression of several cytokines and antiviral genes, which could restrict PRRSV replication *in vitro* (Almeida et al., 2008; Aminul et al., 2017).

Amino acids are required for the synthesis of a variety of specific proteins and the regulation of key metabolic pathways in the immune response to infectious pathogens (Li et al., 2007; Ferrante and Anthony, 2013; Nicoli et al., 2018). In TC pigs, we found that a lot of DEGs and DEPs were involved in the metabolic of amino acids. Amino acids influence cellular metabolism through regulating glycolysis, the TCA cycle, and OXPHOS. Leucine and isoleucine can increase the translocation of the glucose transporters and provide CoA intermediates to the TCA cycle (Kelly and Pearce, 2020). In addition, amino acids could regulate the number of IFN-γ producing cells and the degree of the inflammatory response by providing methyl groups for methylation (Tomé, 2021). An efficient immune response against pathogens is bioenergetically expensive, requiring precise regulation of metabolic pathways (Ganeshan and Chawla, 2014). During PRRSV infection, the host continuously responds to pathogens through innate and adaptive immunity, which carries a considerable bioenergetic, especially in the form of ATP. T cells use glucose, amino acids, and lipids to fuel the TCA cycle and oxidative phosphorylation for ATP production (Fox et al., 2005; Jones and Thompson, 2007; Windt and Pearce, 2012). The genes involved in the TCA cycle were up-regulated in TC pigs, which provides bioenergy for the basic life activities of immune cells. Moreover, NADH, the oxidative product in the TCA cycle, is the critical co-factor required for ROS production in subsequent reaction, which is a key step for the activation of immune cells and also influences the antigen presentation functions (Romero et al., 2016; Lsa et al., 2020).

The activation of cell metabolism and cell cycle are essential for antiviral response (Pujhari et al., 2014). Cell cycle is a highly regulated process to determine the fate of cells, which plays a unique role in controlling lymphocyte proliferation and tolerance (Balomenos and Martínez-A, 2000). The increase of cyclin B and CDK1 complexes in TC pigs could effectively regulate the G2/M phases of the cell cycle and promote the initiation of mitosis (Malumbres and Barbacid, 2005). These pathways were the supplement for the host resistance to PRRSV and may display a broader response, which could be a new approach for antiviral research. Furthermore, the number of samples used in this study is limited, and the regulatory mechanisms of cell metabolism and the antiviral-related pathways need further investigation.

In conclusion, this study integrate transcriptome and proteome datasets to identify DEGs, DEPs and enriched pathways in ILNs between PRRSV-resistant TC pigs and PRRSV-susceptible LW pigs. Our results reveal antigen recognition capacity, immune activation, cell cycles, cell metabolism are important for PRRSV clearance. This study offered huge data for PRRSV research and was helpful for elucidating the mechanism of antiviral research.

## Data Availability

The raw RNA-seq data for this study can be found in the Sequence Read Archive, accession number PRJNA488960.
