# Supplementary material for: Integration of Transcriptome and Proteome in Lymph Nodes Reveal the Different Immune Responses to PRRSV Between PRRSV-Resistant Tongcheng Pigs and PRRSV-Susceptible Large White Pigs
Source: Front Genet. 2022 Jan 27;13:800178. doi: 10.3389/fgene.2022.800178 (PMC8829461; doi:10.3389/fgene.2022.800178)
Supplement: Supplementary file 4 [file DataSheet1.docx]

Supplementary Material

# Supplementary Figures and Tables

## Supplementary Figures


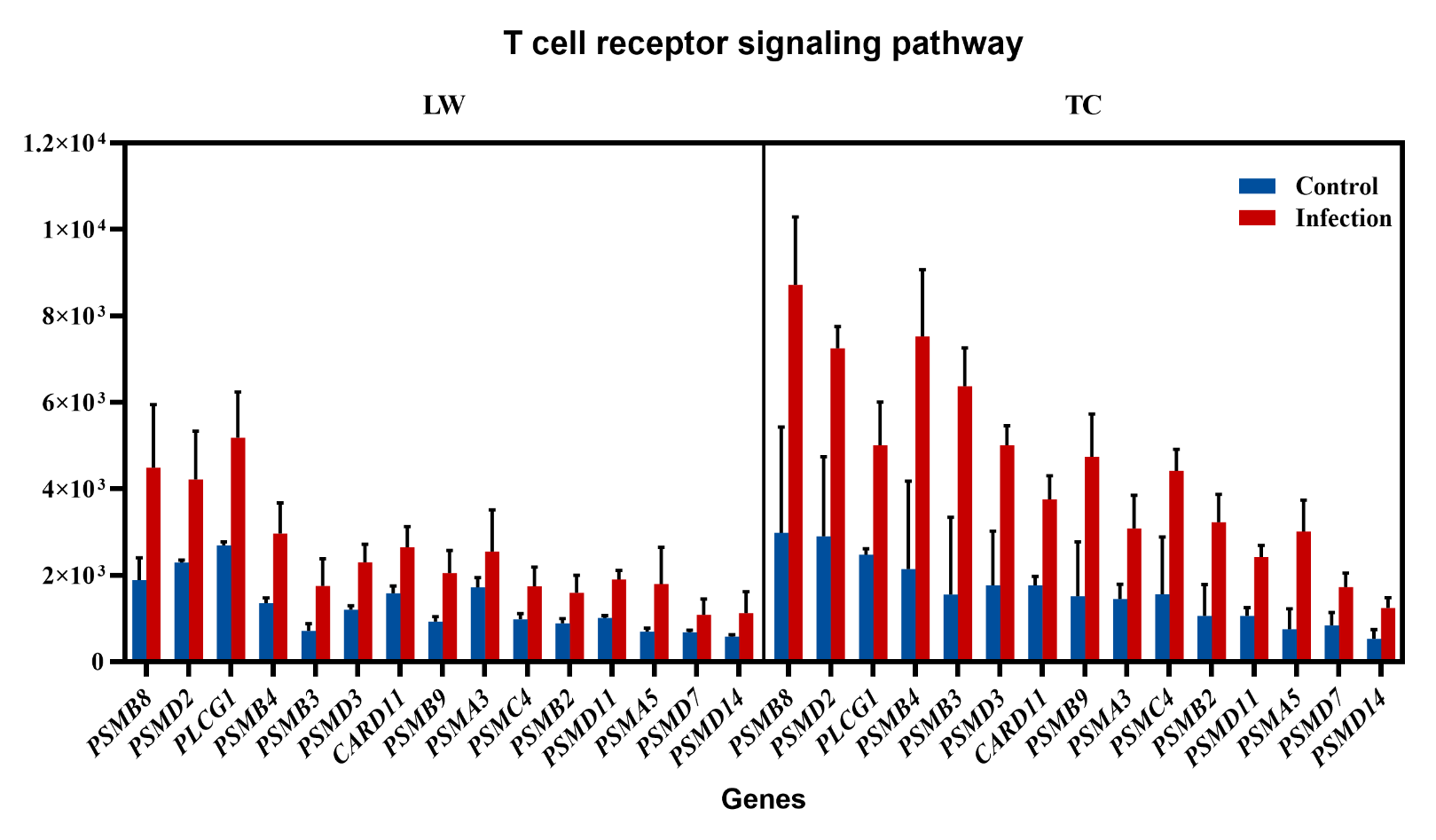


**Supplementary Figure 1.** The expression levels of DEGs in GO enrichment.


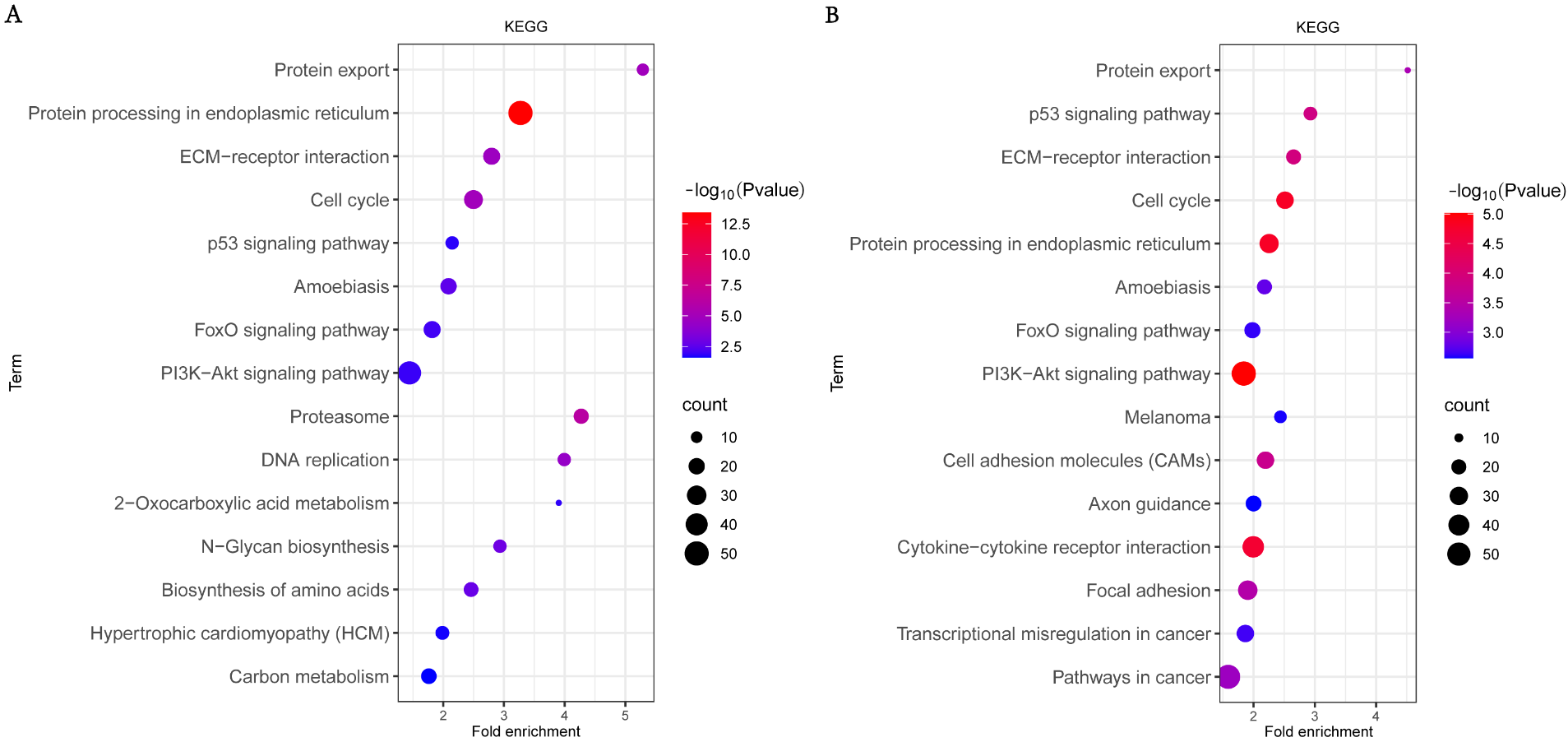


**Supplementary Figure 2.** The KEGG enrichment of the DEGs. (A) KEGG enrichment of the DEGs in TC pigs; (B) KEGG enrichment of the DEGs in LW pigs.


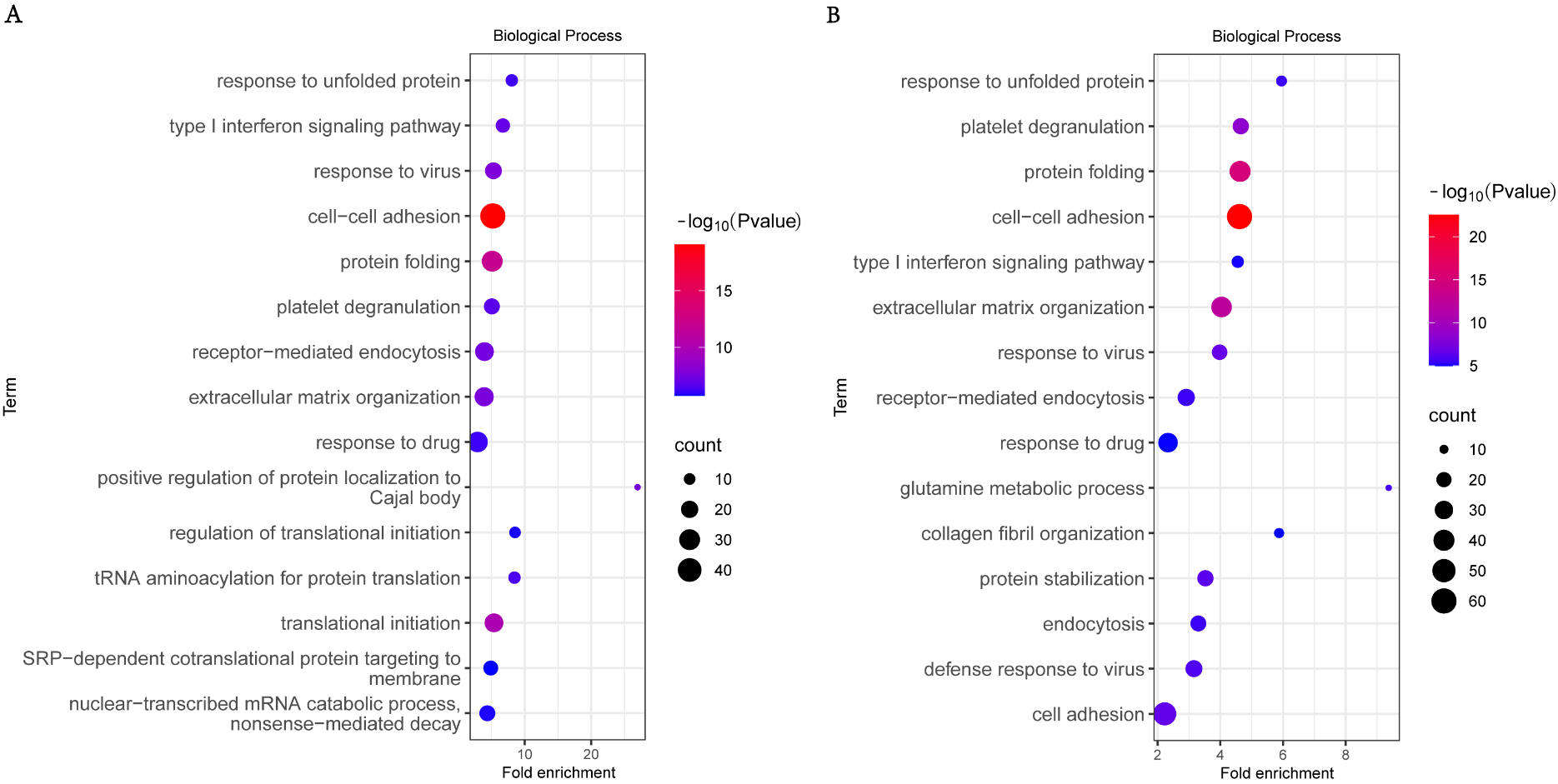


**Supplementary Figure 3.** The gene ontology (GO) enrichment of the DEPs. **(A)** GO enrichment of the DEPs in TC pigs; **(B)** GO enrichment of the DEPs in LW pigs.


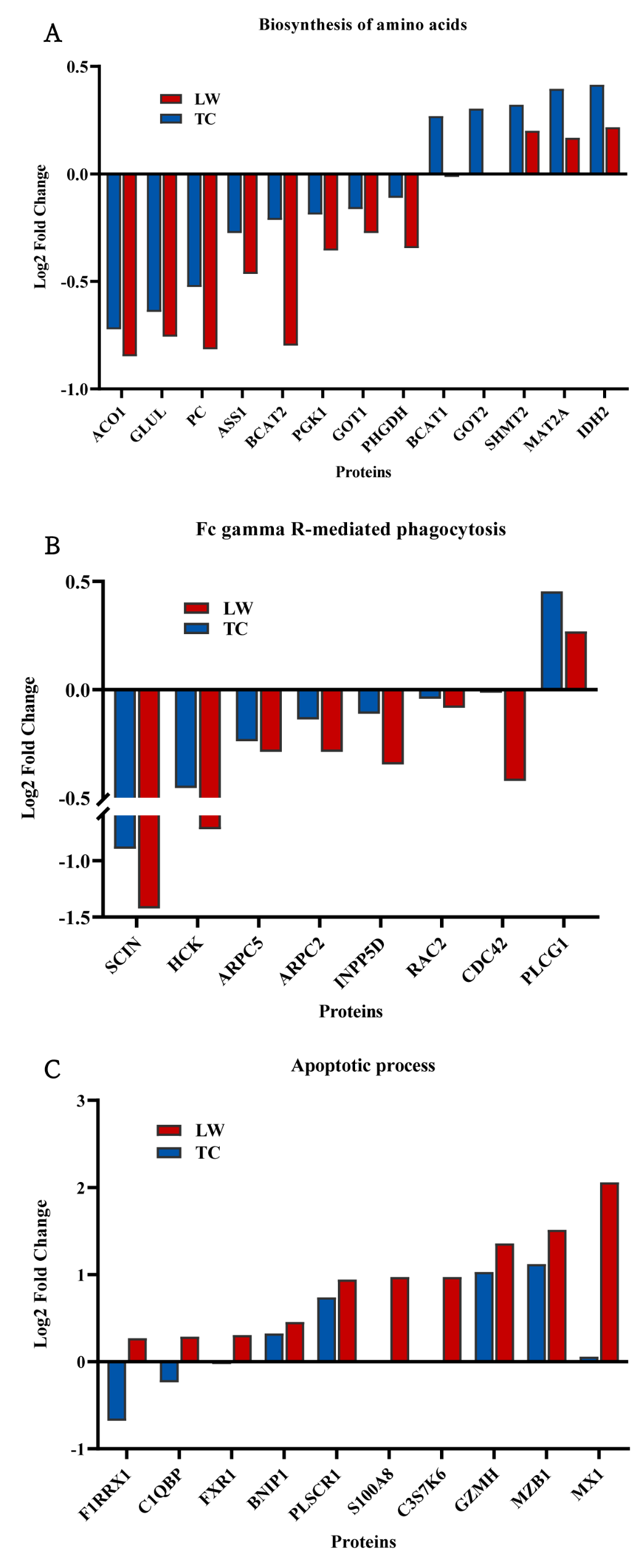


**Supplementary Figure 4.** The abundance of DEPs in TC and LW pigs in different terms. **(A)** DEPs in biosynthesis of amino acids; **(B)** DEPs in Fc gamma R-mediated phagocytosis; **(C)** DEPs in apoptotic process.


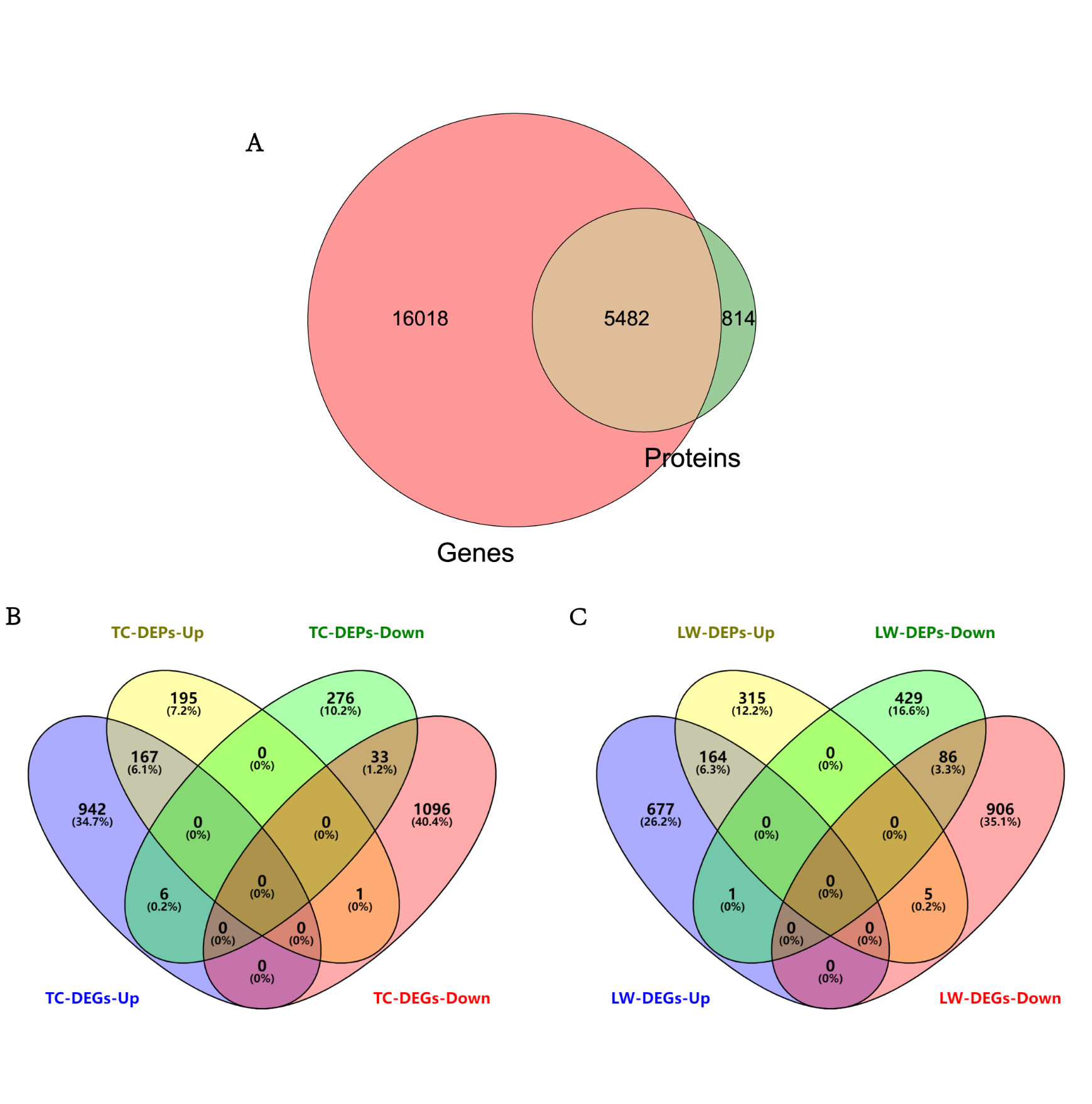


**Supplementary Figure 5.** Venn diagram in TC and LW pigs. **(A)** Venn diagram of genes and proteins; **(B)** Venn diagram of DEGs and DEPs in TC pigs; **(C)** Venn diagram of DEGs and DEPs in LW pigs.

## Supplementary Tables

**Supplementary Table 1.** The detailed information of DEGs.

**Supplementary Table 2.** The detailed information of proteins.

**Supplementary Table 3.** The detailed information of DEPs
